# *Quo vadis* autoimmune hepatitis? - Summary of the 5^th^ international autoimmune hepatitis group research workshop 2024^☆^

**DOI:** 10.1016/j.jhepr.2024.101265

**Published:** 2024-11-12

**Authors:** Bastian Engel, David N. Assis, Mamatha Bhat, Jan Clusmann, Joost PH. Drenth, Alessio Gerussi, María-Carlota Londoño, Ye Htun Oo, Ida Schregel, Marcial Sebode, Richard Taubert, the International Autoimmune Hepatitis Group (IAIHG) collaborators, the European Reference Network for Rare Liver Diseases (ERN RARE-LIVER)

**Affiliations:** 1 Department of Gastroenterology, Hepatology, Infectious Diseases and Endocrinology, Hannover Medical School, Hannover, Germany; 2 Yale School of Medicine, New Haven, CT USA; 3 Ajmera Transplant Centre, University Health Network, Toronto, Ontario, Canada; 4 Else Kroener Fresenius Center for Digital Health, Technical University Dresden, Dresden, Germany; 5 Department of Medicine III, University Hospital RWTH Aachen, Aachen, Germany; 6 Department of Gastroenterology and Hepatology, Amsterdam University Medical Center, The Netherlands; 7 Department of Medicine and Surgery, University of Milano-Bicocca, Monza, Italy; 8 Centre for Autoimmune Liver Diseases & Division of Gastroenterology, Fondazione IRCCS San Gerardo dei Tintori, Monza, Italy; 9 Liver Unit, Hospital Clínic Barcelona, Fundació de Recerca Clínic Barcelona-Institut d’Investigacions Biomèdiques August Pi i Sunyer (FRCB-IDIBAPS), Universitat de Barcelona, Centro de investigación biomédica en red Enfermedades Hepáticas y Digestivas (CIBEREHD), Barcelona, Spain; 10 Liver Transplant and Hepatobiliary Unit, Queen Elizabeth Hospital, University Hospital of Birmingham NHS Foundation Trust & Centre for Liver and Gastro Research, NIHR Biomedical Research Centre, Institute of Immunology and Immunotherapy, University of Birmingham, Birmingham, UK; 11 I. Department of Medicine, University Medical Centre Hamburg-Eppendorf, Hamburg, Germany

**Keywords:** rheumatology, chimeric antigen receptor, artificial intelligence

## Abstract

Autoimmune hepatitis (AIH) is a rare chronic liver disease with an increasing incidence in many countries. Chronic autoimmune responses against the liver can cause hepatic and extrahepatic symptoms, decreased quality of life and reduced liver transplant-free survival if inadequately treated. Although standard treatment with corticosteroids and thiopurines improves the life expectancy of patients with AIH, remission rates and tolerability are generally overestimated and the development of alternative first-line and salvage therapies has been disappointingly slow compared to in rheumatological diseases or inflammatory bowel disease. Other gaps include the lack of disease-specific diagnostic markers for AIH. Similarly, the new entity of drug-induced autoimmune-like hepatitis underscores the need to re-evaluate previous diagnostic criteria. The International AIH Group (IAIHG) has initiated a series of research workshops over the last decade to promote the identification of research gaps and subsequently improve the pace of scientific progress by stimulating collaboration between expert centres. This review reports on the results of the 5^th^ Research Workshop, held in Hannover, Germany in June 2024, and summarises the progress made since the 4^th^ Workshop in 2022. Patient representatives from the European Reference Network (ERN) Rare Liver Youth Panel participated in the workshop. The specific objectives of this year's 5^th^ Workshop were: (1) To further improve diagnostics. (2) Initiate clinical trials including knowledge transfer on drugs from extrahepatic immune-mediated diseases, including B cell-depleting CAR T cells. (3) Utilisation of multi-omics approaches to improve the understanding of disease pathogenesis. (4) Application of machine learning-based approaches established in oncology or transplantation medicine to improve diagnosis and outcome prediction in AIH.

## Graphical abstract

**Figure:**
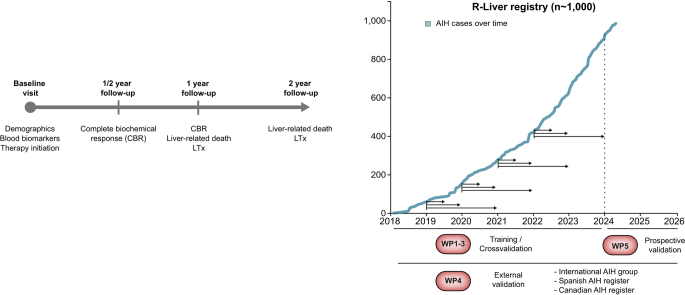

**Keypoints**

• Diagnosing AIH remains challenging due to its diverse clinical presentation and the absence of a single definitive test.
• Polyreactive IgG (pIgG) has emerged as a promising diagnostic marker for AIH, particularly for patients without elevated autoantibody titres, being independent from the presence of classical autoantibodies.
• Emerging options for the improved first-line maintenance treatment of AIH involve the combination of azathioprine and allopurinol or mycophenolate, which has shown promise in improving remission rates and tolerability.
• Future goals include initiating trials for novel (CAR Treg) or repurposed drugs (*e.g.* infliximab, belimumab, CAR T cells), developing a core outcome set for AIH clinical trials to improve quality of life and transplantation-free survival, and establishing comprehensive registries as a synthetic cohort for clinical trials and to document third-line treatments.
• Understanding AIH pathogenesis is limited by the lack of suitable animal models and disease heterogeneity. Recent sequencing technologies have provided new insights into immune dysregulation, particularly in T cell behaviour. However, limited access to liver tissue and the rarity of AIH continue to slow progress in research.
• Usage of AI has been limited in AIH research so far. A machine learning-based approach originating from this years’ IAIHG research workshop will be used to develop a unified approach to prediction of treatment response with the available clinical information (both baseline and longitudinally) and to provide guidance on diagnostic dilemmas.
• AI will be used for pattern recognition in AIH research to help in diagnostic dilemmas (*e.g.* identifying variant syndromes or differentiating AIH and DILI) and, as published in other areas, *e.g.* oncology, will help explain biological differences from H&E stains by linking these with spatial transcriptomics data.

## Introduction

Autoimmune hepatitis (AIH) is a mostly chronic immune-mediated liver disease. The global pooled incidence is estimated at 1.28 cases per 100,000 people whereas the global pooled prevalence is estimated at 15.65 cases per 100,000 people.^1^ Increases in both incidence and prevalence were recently reported.^2^^,^^3^ Patients with AIH often require lifelong immunosuppressive therapy as disease activity is associated with reduced quality of life, as well as liver-related morbidity and mortality.[4], [5], [6], [7] The International Autoimmune Hepatitis Group (IAIHG) is a globally distributed research group comprising experts in the field of AIH, with both clinical and basic scientific backgrounds, that serves as a networking hub to address open research questions and to provide guidance via expert statements on critical issues of AIH research and care.

Unmet needs in AIH were highlighted by previous reports.^8^^,^^9^ Following the previous iteration of the workshop in Maastricht 2022, 65 participants from the IAIHG including paediatric and adult hepatologists as well as patient representatives from the European Reference Network (ERN) Youth Panel met in Hannover (Germany) in June 2024 and paediatric and adult specialists participated in each session. Progress from 2022 to 2024 is summarised in Table 1. The aims of this years’ workshop were to improve diagnostics, initiate clinical trials including transfer of knowledge on drugs from rheumatology, utilise multi-omics approaches to improve our understanding of AIH’s pathogenesis, and develop machine learning-based approaches to improve diagnosis and outcome prediction in AIH. The following conference report is focused on these key issues of the workshop and not an exhaustive review on open gaps in the field of AIH.

**Table 1:** Key issues and outcome after the workshop in 2022.

Key issues	Effort	Outcome
Differentiation of AIH from DILI with autoimmune features	Expert opinion report with agreement on nomenclature and definition of drug-induced autoimmune-like hepatitis^35^ and proposal for new histopathological criteria^118^	Consensus on diagnostic histological for acute and chronic presentation of AIH. Histological criteria retrospectively validated with competing results (In favour of better discrimination: China, multicentre;^119^ UK, single centre;^36^ Italy, single centre;^120^ Turkey, single centre;^121^ Korea, two centres.^122^ In favour of comparable discrimination: Global, multicentre^123^).
Prognosis of AIH with concomitant steatotic liver disease	Retrospective cohort study of patients with AIH with concomitant steatotic liver disease^33^	Coexistence of AIH and steatotic liver disease may impact on overall prognosis of these patients.
Steroid response in acute severe AIH	Retrospective multicentre cohort study^70^ Retrospective multicentre cohort study^124^	AARC score, MELD score, and SURFASA score^125^ accurately identify non-response to steroids at day 3 in patients with acute-on-chronic liver failure. High MELD, presence of encephalopathy or ascites identify patients not likely to respond to corticosteroids.
Non-invasive blood-based markers	Retrospective single-centre cohort study^126^ Retrospective single-centre cohort study^127^ Retrospective multicentre study^25^ Retrospective multicentre studies and prospective single-centre study^27^ Retrospective multicentre study^128^	Quantification of 15 plasma metabolites was highly sensitive and specific to diagnose AIH. Circulating factor H was inversely associated with severity of AIH and likelihood of relapse. Polyreactive IgG was more accurate for the diagnosis of AIH than ANA and anti-SMA in paediatric patients. Polyreactive IgG was more accurate for the diagnosis of AIH than ANA and anti-SMA in paediatric and adult patients with AIH. AMA were present in 5% of AIH-cases but only associated with higher risk for cirrhosis in case of histological bile duct injury.
Liver stiffness measurement	Retrospective multicentre cohort study^129^ Retrospective multicentre cohort study^130^	Liver stiffness measurement was not a good predictor for AIH-related cirrhosis. Higher baseline liver stiffness measurement ≥8.5 kPa was associated with development of cirrhosis and liver-related events.
HCC risk in AIH	Retrospective multicentre cohort study^131^	Low HCC incidence in patients with AIH. HCC associated with obesity, cirrhosis and AIH/PSC variant syndrome.
Recurrence of AIH after LT	Retrospective multicentre cohort study^132^	Younger age at LT, use of MMF post-LT, sex mismatch and high IgG pre-LT associated with AIH recurrence. AIH recurrence is associated with impaired graft and patient survival.
MMF *vs.* azathioprine as first-line therapy	Retrospective single centre cohort study^46^	MMF with higher response rate at 4 weeks and higher rate of CBR at 12 months and at end of follow-up compared to azathioprine.
Tacrolimus *vs.* MMF for second-line therapy in patients not responding to first-line therapy	Randomised-controlled multicentre trial^47^	CBR-rate higher in MMF + prednisolone *vs.* azathioprine + prednisolone for induction therapy. Usage of azathioprine was associated with more adverse events that led to cessation of treatment.
MMF as second-line therapy	Phase IIIB multicentre open-label, parallel-group, RCT^79^ Retrospective single centre cohort study^133^	Recruiting (NCT05221411; EudraCT 2021-003420-33). Approximately 80% response rate if MMF was given for intolerance to first-line therapy but only 30% if given for insufficient response.
Quality of life	Prospective cross-sectional multicentre study^115^	Manuscript currently in revision, published Abstract.
Accuracy of liver biopsy	Retrospective single centre cohort study^135^	Therapeutically relevant differences between left and right lobe liver biopsies in 34% of patients with AIH.^134^
Multiparametric MRI	Prospective single centre cohort study^134^	Increase of iron-corrected T1 over time predicted loss of CBR in patients with AIH that were in remission at inclusion whereas the ELF test and liver sitffness measurement were not predictive.

AARC, APASL ACLF Research Consortium; AIH, autoimmune hepatitis; AMA, antimitochondrial antibodies; ANA, antinuclear antibodies; anti-SMA, anti-smooth muscle antibodies; DILI, drug-induced liver injury; CBR, complete biological response; HCC, hepatocellular carcinoma; LT, liver transplantation; MELD, model for end-stage liver disease; MMF, mycophenolate mofetil; PSC, primary sclerosing cholangitis; RCT, randomised-controlled trial.

## Diagnostic challenges in AIH

Although the first diagnostic criteria for AIH were published about 25 years ago, making an accurate diagnosis remains challenging.^10^^,^^11^ One fundamental issue is that AIH presents with a broad range of severity, from acute liver failure to cirrhosis, sometimes with only mildly elevated liver enzymes and no overt symptoms. No single diagnostic test has been established that is specific for all patients with AIH. While anti-soluble liver antigen/liver pancreas (anti-SLA/LP) autoantibodies are highly specific for AIH, they are present in only a small subset of patients and their positive predictive value is lower in acute liver failures.^12^^,^^13^ Serum IgG, a diagnostic and activity marker, can be negative in at least 10% of cases, especially during the initial presentation of AIH.^14^ Lymphoplasmacytic-rich interface hepatitis is a characteristic histological feature of AIH and is helpful for diagnosis after viral hepatitis has been excluded. However, it is predominantly seen in chronic manifestations of AIH. Acute severe AIH often presents with a more pan-lobular infiltration of immune cells and centrilobular necrosis, frequently lacking typical autoantibodies.[15], [16], [17] As a result, many cases of acute AIH have likely been misclassified as drug-induced liver injury (DILI) in the past.^18^^,^^19^ Therefore, despite their limitations, diagnostic scoring systems remain the best tool for diagnosing AIH. By excluding differential diagnoses that present similarly to AIH and highlighting key serological and histological features, we can narrow down the diagnosis.^20^ This approach is more reliable for chronic presentations than for acute cases, as the diagnostic scores have yet to be validated for the latter.

At the 5^th^ IAIHG research workshop, four key diagnostic challenges were identified and discussed in-depth, leading to the initiation of research projects aimed at addressing these limitations.

### Seronegative AIH

Defining seronegative AIH remains difficult, largely due to variability in test systems, which have different cut-off levels for normal ranges, sensitivities, and specificities for AIH. The two most widely used methods for detecting autoantibodies – indirect immunofluorescence and ELISA – have limited comparability, as the interpretation of immunofluorescence is subjective and dependent on personal experience and test standardisation.[21], [22], [23] Additionally, healthy individuals and patients with other liver diseases, such as steatotic liver disease (SLD) or acute liver failure, may test positive for autoantibodies. For example, up to 20% of patients with SLD may have non-specific autoimmune antibodies, complicating diagnosis.^24^ To mitigate subjective interpretations and variability between test systems, ring trials among reference centres are needed to confirm seronegativity. Moreover, less commonly tested autoantibodies associated with AIH, such as anti-soluble liver antigen/liver pancreas and anti-liver cytosol antibodies, should be screened more frequently to confirm true seronegativity. Technological advances are critical to identify and validate novel diagnostic antibodies with better performance across the broad clinical spectrum of AIH. Since the last IAIHG workshop, polyreactive IgG (pIgG) has emerged as a promising new autoantibody test with higher specificity and overall accuracy for diagnosing untreated AIH compared to conventional autoantibodies, such as antinuclear and anti-smooth muscle antibodies, in both adults and children.[25], [26], [27] pIgGs are independent of conventional autoantibodies and have a similar prevalence in patients with AIH, with or without these autoantibodies. A prospective multicentre study is currently underway to compare the diagnostic accuracy of pIgG with conventional autoantibodies in paediatric and adult patients (NCT05810480). However, it should be noted that pIgG may cause false-positive results in some immunoassays.^22^ Furthermore, serum pIgG levels decrease after the initiation of immunosuppression and, therefore, should be measured before therapy begins. It is hoped that pIgG testing will improve the overall diagnostic process for AIH and potentially increase the detection of cases currently labelled as seronegative AIH.

### Variant syndromes of AIH

Variant syndromes of AIH, which exhibit features of both AIH and cholestatic liver diseases, such as primary biliary cholangitis (PBC) or primary sclerosing cholangitis (PSC), remain challenging to characterise in both clinical practice and research. Although diagnostic criteria have been published to define these variants, uncertainties and controversies persist. For AIH-PBC variants, the Paris criteria have been developed to combine diagnostic criteria for PBC and AIH.^28^ However, there is ongoing debate regarding the components and proposed cut-offs of the Paris criteria. The key issue is determining the extent of hepatocellular inflammation, as indicated by elevated aminotransferase levels, that differentiates AIH from classical PBC. Correctly identifying AIH in patients with PBC allows for the initiation of immunosuppressive treatment to improve outcomes.^29^^,^^30^ However, misdiagnosing AIH variants can lead to inappropriate treatment, particularly with glucocorticoids, which can negatively impact patients’ clinical outcomes and quality of life.^4^

The IAIHG is currently working to improve these diagnostic uncertainties. Prospective studies are needed to assess whether patients with variant syndromes benefit from immunosuppression and whether this treatment can prevent progressive liver damage. A new Delphi consensus process, initiated by the ERN RARE-LIVER in collaboration with IAIHG, aims to better define AIH-PBC variants, with updated diagnostic criteria expected to be published in late 2024 or early 2025. A similar process for AIH-PSC variants is anticipated to begin in 2025. The IAIHG remains committed to addressing the unmet diagnostic needs for variant syndromes, and the results of these initiatives will be presented at the next IAIHG workshop in 2026.

### AIH and SLD

The differential diagnosis between AIH and SLD, and the possible concurrence of the two conditions, presents a significant clinical challenge. The increasing incidence of SLD, including its subtypes such as MASLD (metabolic dysfunction-associated steatotic liver disease) and MASH (metabolic dysfunction-associated steatohepatitis), complicates AIH diagnosis due to the frequent presence of non-specific autoantibodies in patients with SLD.^31^ A recent histological analysis of patients with advanced MASH further highlighted this challenge, as interface hepatitis and plasma cells were commonly observed.^32^ Although case series on the co-occurrence of AIH and SLD have been published, long-term prospective analyses are lacking.^33^^,^^34^ Key questions remain: Does the coexistence of AIH and SLD lead to a more aggressive disease course? How does steatohepatitis influence the autoimmune inflammation of AIH? To what extent do steroids used to treat AIH, such as budesonide or systemic steroids, exacerbate or induce SLD? Additionally, do AIH diagnostic scores need to be adjusted when SLD is suspected? How can we define biochemical remission in case of coexisting metabolic inflammation?

### AIH *vs*. DILI

Distinguishing AIH from DILI, particularly when DILI mimics AIH with elevated aminotransferases, autoantibodies, and immune cell infiltration, remains a diagnostic challenge. This subtype of DILI, referred to as drug-induced autoimmune-like hepatitis (DI-ALH), was recently defined.^35^ Since no adequate diagnostic tools are available at the time of presentation, the best approach to differentiate AIH from DILI involves monitoring the clinical course over time. AIH is likely to relapse after tapering immunosuppression, while DI-ALH typically does not. Most centres administer steroids during acute presentations and then gradually reduce the dosage, holding off on initiating long-term steroid-sparing agents. If liver enzymes rise again, AIH is considered the final diagnosis, and maintenance therapy is started. However, this process, which relies heavily on temporal monitoring, poses a burden for both patients and clinicians. Regular follow-up is essential, as AIH activity can spontaneously decrease and relapse after several years. Without systematically attempting to withdraw steroids or immunosuppressive therapy within the first few months, distinguishing AIH from DI-ALH is difficult in patients without advanced fibrosis.

The IAIHG workshop emphasised the need for a multicentre investigation, in collaboration with expert DILI groups, to improve our understanding of cases where AIH *vs.* DI-ALH is suspected. This investigation would involve a standardised protocol for collecting baseline clinical data, administering immunosuppression, withdrawing therapy at a predefined time, and conducting regular follow-up. Additionally, biobanking of clinical specimens could help identify novel diagnostic biomarkers.

### Future goals/actionable items

• Autoantibodies:
  ○ Perform a retrospective multicentre study on the diagnosis of seronegative AIH in all age groups, which is being initiated within the IAIHG.
  ○ Perform a prospective multicentre study to validate the diagnostic accuracy of pIgG in a head-to-head comparison with conventional autoantibodies in adults and children, which is currently recruiting (NCT05810480).
• Liver diseases overlapping with AIH:
  ○ Complete the Delphi consensus process started by the IAIHG and ERN RARE-LIVER to better define AIH-PBC variants, with updated diagnostic criteria expected to be published in late 2024 or early 2025.
  ○ Begin a similar process for AIH-PSC variants in 2025.
  ○ Collaborate with experts in the SLD field to develop a joint clinical research strategy addressing questions on concomitant AIH and SLD, which was initiated during the workshop.
• AIH, DILI, DI-ALH:
  ○ Standardise protocols for collecting baseline clinical data, administering immunosuppression, withdrawing therapy and longitudinal supervision to improve diagnostic criteria, distinguish between entities at baseline, and further develop the current consensus through collaboration of the IAIHG and PRO-EURO-DILI network.^35^
  ○ Continue subprojects focusing on various aspects of differentiating AIH, DILI and DI-ALH, as initiated by members of IAIHG and PRO-EURO-DILI, with results due to be reported at the next meeting in 2026.
  ○ Reclassify existing databases/registries and biorepositories to determine whether or not AIH and DI-ALH can be distinguished retrospectively.^36^

## Treatment of AIH

### What can we learn from treatment of other immune-mediated diseases?

First-line therapy for AIH is still limited to corticosteroids (mostly predniso(lo)ne, budesonide) and thiopurines (mostly azathioprine, alternatively 6-mercaptopurine).^37^^,^^38^ Recent advances include evidence-based recommendations for therapeutic drug monitoring (TDM) of azathioprine and optimisation of its metabolism into the immunosuppressive metabolite 6-thioguanine-nucleotide with the addition of allopurinol to increase remission rates and tolerability, as in inflammatory bowel disease (IBD).[39], [40], [41], [42] Additionally, recent real-world experience with budesonide in Spain did not fully confirm the promising results of the randomised-controlled trial (RCT).^43^^,^^44^ Mycophenolate mofetil (MMF), which is most commonly used after organ transplantation, is still recommended as second-line therapy in AIH for incomplete remission and intolerance on standard therapy,^37^^,^^45^ but the demonstration of higher biochemical remission rates and much better tolerability compared to azathioprine within the first year challenges current recommendations.^46^^,^^47^ However, teratogenicity is a disadvantage of MMF compared to azathioprine. Long-term results of MMF monotherapy and combination therapies are eagerly awaited. Considering improved response rates to the combination of low-dose azathioprine with allopurinol compared to azathioprine monotherapy,[39], [40], [41], [42] a head-to-head comparison of this combination (with TDM) against MMF would be of interest.

Regarding third-line therapy in cases of non-response to corticosteroids, azathioprine and/or mycophenolate, B-cell depletion is a common therapeutic approach in rheumatological diseases. Published experiences of rituximab in AIH are limited to retrospective multicentre studies showing improved disease activity with low rates of infectious complications.^48^^,^^49^ Incomplete responses with rituximab could be related to complement-dependent cytotoxicity and reduced depletion in liver tissue.^50^ In contrast, belimumab, an antibody against soluble BAFF (B cell-activating factor), showed less promising results in a small case series.^51^ B-cell depletion via chimeric antigen receptor (CAR) T cells or bispecific T cell engagers, originally developed for the treatment of B-cell malignancies, showed promising results for advanced and refractory rheumatological autoimmune diseases by inducing a very deep B-cell depletion in blood and tissue, mediating a reset of the B-cell repertoire without severely impacting humoral immunity.[52], [53], [54], [55], [56]

Due to overlap of AIH with extrahepatic autoimmune diseases, limited experience with immunomodulatory drugs was published recently.^51^ These limited experiences are just a starting point for further data collection, *e.g*. within the R-LIVER registry of ERN RARE-LIVER (https://rare-liver.eu/healthcare-professionals/r-liver-registry).

Beyond the budesonide study,^57^ none of the aforementioned therapeutic studies in AIH included children. This underlines the urgent clinical need to include children in studies on new treatment approaches.

### Future goals/actionable items

• Report experiences with immunomodulatory drugs given to patients with AIH to treat coexisting autoimmune diseases (*e.g.* IBD, rheumatologic diseases, …) in all age groups.
• Report on salvage therapies irrespective of their success, preferably within registries over all age groups.
• Compare azathioprine/allopurinol combination therapy (with TDM) to MMF as first-line therapy.
• Initiate trials with new or repurposed drugs as first-line therapy in AIH in adults and children.

### Cell therapy approaches

So far, cell therapies, beyond the most recent B-cell depletion via CAR T cells, have aimed at tipping the immunological balance towards hepatic tolerance by strengthening regulatory T cells (Treg), *e.g.* by reinfusion of polyclonal autologous Tregs after apheresis and *in vitro* expansion. Since a first pilot study demonstrated the safety of this approach and a recirculation of these expanded polyreactive Tregs into the liver and spleen,^58^ a follow-up study on Treg infusion in patients with PSC and IBD demonstrated the clinical efficacy of this approach.^59^ Treg therapy in PBC is still in progress. Another approach is the *in vivo* simulation of Tregs by adding low dose interleukin 2 (IL-2), which showed some success in a small case series of patients with AIH.^60^ However, a low dose IL-2 supplementation did not promote tolerance but rather caused rejection during withdrawal of immunosuppression after liver transplantation,^61^ showing the narrow range of IL-2 supplementation between stimulating Tregs and effector T cells.^62^ Another approach used the CAR technology combined with transduction of *FOXP3*, a key transcription factor of Tregs, to generate an antigen-specific so-called CAR Treg. In proof of concept studies, these CAR Tregs were shown to improve control of allo- and autoimmunity in animal models of solid organ transplantation and experimental autoimmune diseases.[63], [64], [65], [66], [67], [68] However, regulatory hurdles and the high requirements for GMP facilities to produce such cell products will dictate the speed of progress in this field.

### Future goals/actionable items

• Create a suitable environment for immune regulation, including by applying anti-proinflammatory cytokine therapy for pre-conditioning.
• Apply combination or sequential cytokine and regulatory cell therapy.
• Treat patients in the early stages of disease when there are less tissue resident and effector memory cells.
• Stratify patients by the relevant pathogenic pathways using omics and immunophenotyping to determine whether T cells or B cells or cytokine-directed therapy are optimal.

### Clinical trials

Therapeutic recommendations for AIH traditionally depend on single-arm observational studies as the AIH field is devoid of clinical trials.^69^ Recent observational studies highlight a clear clinical need^44^^,^^49^^,^[70], [71], [72], [73]): only 27% of all patients with AIH achieve a steroid-free remission within the first year, and over 30% experience drug-related side effects within 6 months after treatment initiation.^74^ Notably, patients prioritise better disease control and safety, and are willing to participate in clinical trials.^4^^,^^75^^,^^76^

The CAMARO study published in 2024, which examined azathioprine *vs.* MMF as first-line therapy, was the first RCT published since 2010.^43^^,^^47^ Ongoing trials include the AIH-MAB phase IIa, proof-of-principle study exploring infliximab, with limited data available in AIH,^77^^,^^78^ for remission induction (EudraCT: 2017-003311-19) and the AMBER (Antibody-dependent cellular cytotoxicity (ADCC) Mediated B-Cell depletion and BAFF-Receptor Blockade) study (NCT03217422) which completed recruitment in 2023 with results yet to be published.

The only RCTs currently recruiting are a phase II study evaluating the efficacy and safety of HR19042 (NCT05476900) and the TAILOR trial, a phase IIIB multicentre, open-label, parallel-group RCT comparing tacrolimus and MMF in patients with AIH not responding to first-line therapy.^79^ The BELief Study, a multicentre, open-label trial to examine belilumab, a B-cell activating factor inhibitor, will begin recruiting in 2025 (NCT06381453).

What do we need to consider when designing a clinical trial in AIH? Key factors are selection of drugs (novel or repurposed), targeted treatment phase (induction or remission), clear inclusion and exclusion criteria and well selected outcomes. Additionally, funding and multicentre collaboration are crucial.^51^^,^^80^

The selection of drugs to test in a clinical trial is central to its success and prior documented experience in AIH helps to estimate the chance of success. To facilitate documentation of third-line treatments, a dedicated registry within the ERN RARE-LIVER’s R-LIVER, is expected to launch in 2024.

A core outcome set for clinical trials, as defined for other autoimmune liver diseases (see^81^), is absent and represents an urgent need. Possible endpoints of clinical studies include: (1) improvement of symptoms, (2) biochemical remission according to the normalisation of alanine aminotransferase, aspartate aminotransferase, and IgG, (3) histological remission with a modified histological activity index) ≤3.^37^^,^^38^^,^^82^^,^^83^ However, these biomarkers represent intermediate endpoints, hence therapeutic options must improve quality of life (QoL) and transplantation-free survival.^76^^,^^84^

Several, but not all, recent studies have questioned the need for IgG normalisation, because there is no association between IgG levels and disease progression.^6^^,^^7^^,^^14^^,^^71^^,^^85^^,^^86^ Combining alanine aminotransferase with IgG slightly improves accuracy, and a meta-analysis on the relevance of IgG normalisation for hard treatment endpoints is the next step.^74^^,^^83^^,^^87^

The generation and validation of synthetic cohorts that replicate patient outcomes under control treatments would be highly beneficial. Multicentre data sets are available and published, including the IAIHG retrospective registry,^6^ national registries^7^^,^^44^ and the prospective AIH registry of the ERN RARE-LIVER (R-LIVER).^74^ However, to promote the comprehensiveness of these data sets, these registries should expand to include safety data for the drugs patients are exposed to. A validated questionnaire that assesses QoL in AIH remains a major unmet need.^4^^,^^72^^,^^88^

Recruitment in clinical trials is hindered by rigid inclusion and exclusion criteria. We favour a pragmatic approach that expands the recruitment criteria while keeping safety and expected therapeutic effects in balance. We encourage the AIH field to abandon the contention of therapeutic nihilism, assuming current treatments are sufficient despite their suboptimal efficacy and safety. To spur recruitment, the IAIHG plans to publish a dashboard highlighting these multicentre studies for patients of all age groups.

### Future goals/actionable items

• Perform a meta-analysis and prospective registry analysis on prediction of outcome with parameters of biochemical remission (normalisation of transaminases with and without IgG, time to achievement of remission), which has already been initiated following this years’ workshop (also see AI section), for patients of all age groups and all AIH manifestations.
• Update consensus documents regarding treatment aims based on these new results.
• Establish a study dashboard for multicentre studies on the IAIHG homepage.
• Perform a structural and qualitative expansion of the existing registry to provide the basis for a quality-controlled synthetic cohort including aspects of safety, QoL and patient-reported outcome measures.
• Continue the consensus process on a core outcome set and standard of care in clinical trials which was initiated during the workshop.

## Pathophysiology and basic science

Several obstacles have hampered progress in understanding the pathogenesis of AIH, including the lack of an animal model that completely recapitulates the persistent loss of tolerance to liver antigens observed in clinical settings, and the tremendous heterogeneity of the disease with diverse environmental triggers, different genetic backgrounds, various clinical phenotypes and manifestations in different age groups. This could contribute, at least in part, to the controversial results observed in previous studies regarding the number and function of specific effector and regulatory cells in patients with AIH and their role in the perpetuation of intrahepatic immune system activation.

Scientific and technological advances in life sciences are intrinsically linked, and most recent advances in sequencing methodologies such as single-cell RNA sequencing, single-nucleus sequencing and tissue spatial transcriptomics such as CosMx™ and Xenium have significantly enhanced our understanding of pathophysiology. However, the number of publications that have applied these new novel technologies in the field of AIH, especially in human AIH, remains limited.[89], [90], [91], [92], [93], [94] Nevertheless, these studies have yielded noteworthy results: 1) autoreactive TCR (T cell receptor) clonotypes were predominantly found in antigen-specific CD4+ T cells exhibiting a memory phenotype with characteristics of peripheral helper T cells^90^; 2) peripheral and intrahepatic T cells demonstrated a TRBV-J-biased signature that persisted after effective immunosuppressive treatment, suggesting that immunosuppressive treatment affects T cell function rather than the underlying T cell architecture, potentially explaining the high relapse rate in AIH^91^; 3) upregulated genes in peripheral mononuclear blood cells from patients with AIH were primarily derived from monocytes and NK cells and participated in pathways related to antigen processing and presentation, and interferon-gamma signalling^89^; and 4) hyperexpanded TCR clonotypes were predominantly observed in CD8+ T cells of patients, indicating a significant role of these cells in antigen recognition in AIH.^94^ There are, however, significant limitations that likely account for the relatively slow progress in utilising these technologies to elucidate the mechanisms underlying immune system dysregulation in AIH. Firstly, AIH is a rare disease and funding opportunities are limited, particularly for early-career investigators. Secondly, regulation of immune cells and cytokines in the peripheral blood and in the liver itself is divergent,^95^ which mandates the use of liver samples to comprehensively understand the changes in the intrahepatic immune microenvironment. However, access to liver tissue is obviously challenging, especially longitudinally under therapy. Consequently, alternative approaches have been explored, and although fine needle aspiration samples are not entirely representative of the intrahepatic milieu, they appear to offer a practical solution.^95^ The utilisation of liver explants is another option that provides sufficient liver tissue. However, its use is restricted to cases of acute liver failure or end-stage liver diseases and therefore, the results from these samples are likely not representative of disease heterogeneity.^96^^,^^97^ The clinically available liver tissue from core needle biopsy cylinders might be insufficient for the analysis of rare immune cell types (*e.g*. Tregs) or for digital spatial transcriptomics approaches. Finally, special populations of patients with AIH such as children or pregnant women have rarely been included in these studies and knowledge around the immunological changes in these patients is very limited.

### Future goals/actionable items

• Nurture future leaders through mentoring strategies within the framework of international societies and interest groups.
• Foster collaboration and data integration across ERN RARE-LIVER and IAIHG.
• Prepare a protocol to unify and standardise sample collection that can be shared among centres with different expertise, which was initiated at the workshop.
• Link paediatric and adult cohorts for longitudinal study, a joint meeting together between ESPGHAN and EASL is desirable.
• Propose a monothematic meeting on AIH within the EASL school of hepatology framework.
• Obtain funding for cross EU collaboration with Marie Curie type programme.

## Artificial intelligence

The usage of AI (artificial intelligence) has drastically increased over the past years across various scientific disciplines in medicine, *e.g*. in oncology and histopathology,^98^ or after liver transplantation.^99^ Models of various complexity – ranging from comparably simple and explainable machine learning (ML) algorithms, to more complex neural network architectures for image analysis and, most recently, large language models (LLMs) – have shown promise in medicine with potential for application in AIH research and healthcare. ML-based approaches can inform physicians about patients’ trajectories in terms of outcome by incorporating longitudinal patient data.^100^ Deep learning can aid in identifying disease phenotypes or predicting molecular markers from H&E-stained histological slides.^101^ LLMs can empower patients and increase guideline adherence.^102^^,^^103^ Within the field of autoimmune liver diseases, a recent publication firstly shows that a transformer-based deep learning model can differentiate AIH and PBC using H&E-stained slides, and outperforms resident pathologists in diagnosing the respective diseases.^101^ However, as with any other biomarker, the role of AI for diagnosis, prediction, and decision support in AIH will have to be evaluated rigorously and validated prospectively in a multicentre fashion to assure its universal applicability in the targeted clinical setting, in particular both adult and paediatric patients have to be evaluated for tools to be applicable in relevant real-world clinical scenarios. However, in contrast to oncology, for example, many topics with potential for AI and ML-based solutions remain unaddressed within the field of AIH. That is why this year’s iteration of the IAIHG research workshop had a specific focus on AI-based approaches aiming to deliver specific objectives to address in AIH research until the next workshop takes place. In this regard, the 5^th^ IAIHG research workshop stated their focus on interpretability, reproducibility, and generalisability of any model in AIH, which are crucial for final clinical implementation.

### AI and clinical data

**Fig. 1:**
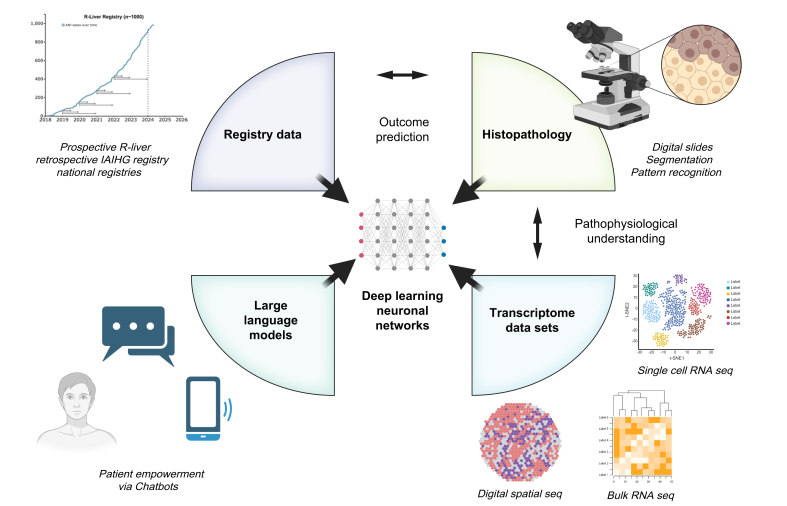
Research areas for AI approaches in AIH. AI, artificial intelligence; AIH, autoimmune hepatitis.

A particular strength of the closely collaborating community of AIH researchers lies in the establishment of prospective, multicentric cohorts like the R-LIVER registry, the retrospective registry of the IAIHG, as well as national registries.^6^^,^^7^^,^^44^^,^^74^ The comprehensive data in these registries, including demographics, treatment regimens, blood data and much more can serve as the basis for ML-based predictive models. Prediction of treatment response is essential in AIH treatment as normalisation of transaminases and IgG (complete biochemical response [CBR]) correlates with long-term outcomes.^6^ Hence, achievement of CBR at 6 months was determined a goal of therapy by the IAIHG,^83^ which has recently been externally validated.^104^ Further prediction of CBR at 12 months, prediction of liver-related events, liver transplantation or death are essential for a more personalised approach to treatment of AIH. Further, it could be possible to detect subgroups at higher risk of treatment failure early-on, as shown in PBC.^105^ A clear agreement among participants of the 5^th^ AIH workshop was that baseline data and longitudinal trajectories should be integrated into ML-based models to predict the respective endpoints. Building on this position, the instructed taskforce already applied for joint data access to all four previously mentioned registries, with the project proposal outlined in Fig. 1, consisting of a phase of model training, external validation and prospective validation. If successful, this method could serve as a blueprint for other rare liver diseases.

### AI and histology

Liver biopsy is essential in diagnosing AIH, yet the wealth of information embedded within liver slides is still largely underutilised. Histological data are rarely incorporated into prognostic models or used to guide treatment regimens. Furthermore, at the diagnostic level, liver biopsy assessments remain largely semi-quantitative, if not entirely qualitative, which can lead to diagnostic errors, with potential harmful consequences for patients. The application of computational pathology could significantly address these limitations.

A recent collaborative multicentre study from Europe demonstrated that semi-supervised deep learning can accurately distinguish AIH from PBC and outperforms non-specialist pathologists.^101^ Building on this work, participants at the 5^th^ AIH Workshop agreed to collaborate on creating a pathological atlas of PBC, AIH, and DILI cases. This effort aims to validate the initial findings and address unmet needs, such as diagnosing the PBC-AIH variant syndrome and DI-ALH. One major challenge for this ambitious project is overcoming regulatory barriers to data sharing across countries. Swarm learning, which has shown similar accuracy to centralised approaches,^106^ may offer a solution by facilitating easier data sharing. A medium-term goal for the group is to establish local IT infrastructures to participate in the existing swarm learning network developed along the PBC-AIH differentiation project,^112^ enabling safe and effective project execution.

In other fields, particularly oncology, scientific evidence has been used to link H&E images to AI-generated heatmaps and gene expression patterns derived from spatial transcriptomics.^107^ An important objective of this initiative is to generate spatial transcriptomics data for a subset of patients to better understand the biological pathways in areas identified by AI as relevant for diagnosis and prognosis.

Currently, histological and clinical data analysis remain separate entities. Another key project will focus on integrating histological data, derived from deep learning analyses, with clinical data to determine whether the addition of histological information can improve risk stratification at the time of diagnosis.

### AI-generated patient information

Patients have free access to AI tools like ChatGPT and have access to AI-generated patient information. The safety of such patient information is a matter currently being investigated within the IAIHG. A very recent study highlighted areas of improvement of chatbots’ answers with regard to the management of patients with autoimmune liver diseases and identified areas for improvement.^102^

It was also discussed how AI tools can be trained on large datasets to integrate several different modes of data (clinical, laboratory and radiomic variables, among others), longitudinal changes and patterns over time, to provide personalised clinical predictions.^108^^,^^109^ Individual risk factors, both modifiable and non-modifiable, predictive of those personalised predictions can be identified. Such AI tools can be deployed via electronic medical record systems to provide such individualised predictions, which can guide precision care.^110^ Such integration of multiple variables into individualised diagnosis and therapeutic recommendations would be ideal for the future care of patients with AIH, pending the first AI-based analyses and subsequent validation studies, as outlined above.

### Guideline adherence

It is challenging to enforce guideline therapy in rare diseases such as AIH. This is mostly due to lack of awareness and familiarity with the recommendations, as well as lack of domain knowledge.^111^ LLMs have been shown to excel at guideline-adherent treatment recommendations when combined with concepts such as retrieval-augmented generation.^103^^,^^112^ Here, the LLM does not provide an answer based on its training data, but rather bases its answer on explicitly provided data, such as a collection of guidelines on all rare diseases in hepatology, drastically reducing hallucinations. Applying LLMs as rare-disease-specialists for AIH could help general practitioners with earlier diagnosis in settings where no AIH specialist is available, and to overall improve guideline adherence.^113^

### Future goals/actionable items

• Follow an AI-based analytic approach to unify currently available registry data for the prediction of CBR, which was initiated at the workshop.
• Continue projects focusing on AI-based analysis of histopathological slides with a focus on difficult diagnostic situations as well as prediction of biological patterns in H&E slides with data derived from spatial transcriptomics.
• Develop and validate LLMs, which may support guideline-adherent decision making as well as inform patients longitudinally about their disease trajectories and help to increase patient adherence, once their accuracy has been validated in the field of AIH.

## Patients

Current guidelines recommend an assessment of QoL in patients with AIH^37^^,^^38^ and previous single-centre or national multicentre studies reported a reduced QoL in patients with AIH.^4^^,^^72^^,^^88^^,^^114^ QoL was not a specific focus of the current research workshop but assessment of QoL in an international study was a future goal of the 4^th^ workshop in 2022. The results of this study including over 800 patients with AIH from 12 European centres confirmed the reduced QoL^115^ and the publication is currently in revision.

Patient representatives from the ERN Rare Liver Youth panel participated in the 5^th^ research workshop and presented their individual experience of their personal journey. Thereby, the wish for more understandable and empathic communication from healthcare workers, especially towards young patients, was expressed by both representatives, mirroring the results of a recent international European study.^116^ Ultimately, better communication with patients, including patient educational programmes aimed at patients with AIH, may help improve disease understanding and increase patient adherence, as already shown in inflammatory and immunologic diseases.^117^

## Conclusion

The IAIHG research workshops have been established as a regular forum for experts in the field that also allow for patient participation. Considering the time required for prospective or retrospective multicentre studies, including the subsequent publication process, not all actionable items can be fully addressed within the 2 years until the next workshop. Therefore, new focus areas, such as rheumatologic therapies, multi-omic approaches and AI methods in the current workshop, need to be included to promote knowledge transfer from other areas of research and medicine. However, both the initiation and continuation of multicentre studies benefit from focused workshops outside of major conferences. So far, all workshops have taken place in Europe and have been dominated by European centres and adult hepatologists and AIH researchers. Future workshops could benefit from a more balanced representation of all continents and more intense collaboration with the paediatric field. Measures from funding bodies to facilitate the participation of representatives from low-income countries would be desirable.

## Abbreviations

AI, artificial intelligence; AIH, autoimmune hepatitis; CAR, chimeric antigen receptor; CBR, complete biochemical response; DILI, drug-induced liver injury; DI-ALH, drug-induced autoimmune-like hepatitis; ERN, European Reference Network; HCC, hepatocellular carcinoma; IAIHG, International Autoimmune Hepatitis Group; IBD, inflammatory bowel disease; IL-2 - Interleukin-2; LLMs, large language models; ML, machine learning; MMF, mycophenolate mofetil; PBC, primary biliary cholangitis; pIgG, polyreactive immunoglobulin G; PSC, primary sclerosing cholangitis; QoL, quality of life; SLD, steatotic liver disease; TDM, therapeutic drug monitoring; Tregs, regulatory T cells.

## Financial support

10.13039/501100001659German Research Foundation, Germany (project number 543100176), YAEL Foundation, 10.13039/100001610Falk Foundation, Germany and Association for further training in the GHE e.V. provided the funding to host the IAIHG research workshop in June 2024 in Hannover. BE was supported by the PRACTIS – Clinician Scientist program of 10.13039/501100005624Hannover Medical School, Germany, funded by the 10.13039/501100001659German Research Foundation (DFG, ME 3696/3). JC is supported by the Mildred-Scheel-Postdoktoranden programm of the 10.13039/501100005972German Cancer Aid, Germany (grant #70115730). AG was supported by the following grants: Italian MUR 10.13039/100017336Dipartimenti di Eccellenza, Italy 2023–2027 (l. 232/2016, art. 1, commi 314–337), Italian MUR PNRR PE06 “HEAL ITALIA – Health Extended Alliance for Innovative Therapies, Advanced Lab-research and Integrated Approaches of Precision Medicine" - Spoke 4 - Precision Diagnostics, Italian MUR PRIN 2022 PNRR P2022H7JYZ “Non-invasive biological and molecular characterisation of autoimmune liver diseases and variant syndromes''. Ye Htun Oo was funded by Sir Jules Thorn Biomedical Research Programme Award and Whitney Wood Fellowship from 10.13039/501100000395Royal College of Physicians, United Kingdom.

## Authors’ contributions

Conceptualization and supervision: BE, RT. Writing - original draft: all authors. Writing - review & editing: all authors. Designed the figure: JC, BE, RT with Biorender. Generative AI was used in the preparation of this manuscript and all content was checked for correctness afterwards.

## Conflicts of interests

RT is co-inventor of a patent for polyreactive IgG for the diagnostic of AIH (EP3701264 B1; US 12,044,682 B2). RT and BE received consumables from Inova Inc. and Euroimmun Medizinische Labordiagnostik AG free of charge for other projects. BE received funding from the MHH Förderstiftung Plus to initiate a prospective multicentre trial on the role of polyreactive IgG for the diagnosis of steroid dependent hepatitis. IS received financial compensation for a lecture sponsored by Falk. The other authors report no conflicts of interest related to this work.

Please refer to the accompanying ICMJE disclosure forms for further details.
